# Biochemical and Genetic Engineering of Diatoms for Polyunsaturated Fatty Acid Biosynthesis

**DOI:** 10.3390/md12010153

**Published:** 2014-01-07

**Authors:** Hong-Ye Li, Yang Lu, Jian-Wei Zheng, Wei-Dong Yang, Jie-Sheng Liu

**Affiliations:** Key Laboratory of Eutrophication and Red Tide Prevention of Guangdong Higher Education Institutes, Jinan University, Guangzhou 510632, China; E-Mails: biglvyangaa@gmail.com (Y.L.); jwzheng89@126.com (J.-W.Z.); tywd@jnu.edu.cn (W.-D.Y.)

**Keywords:** diatom, fatty acids, PUFA, genetic engineering

## Abstract

The role of diatoms as a source of bioactive compounds has been recently explored. Diatom cells store a high amount of fatty acids, especially certain polyunsaturated fatty acids (PUFAs). However, many aspects of diatom metabolism and the production of PUFAs remain unclear. This review describes a number of technical strategies, such as modulation of environmental factors (temperature, light, chemical composition of culture medium) and culture methods, to influence the content of PUFAs in diatoms. Genetic engineering, a newly emerging field, also plays an important role in controlling the synthesis of fatty acids in marine microalgae. Several key points in the biosynthetic pathway of PUFAs in diatoms as well as recent progresses are also a critical part and are summarized here.

## 1. Introduction

Marine diatoms have been explored recently as sources of secondary metabolites with biological activity. Diatoms are known to be a reserve source for biofuel, marine drugs, and live feed, owing to their short life cycle, fast growth and simple nutritional requirements [1,2]. Marine diatoms contribute to approximately 40% of primary productivity in marine ecosystems and 20% of global carbon fixation [1,3]. Diatoms are not only photosynthetic autotrophs but can also be symbiotic with photosynthetic organisms [4,5]. Diatoms are considered to be highly diverse, with an estimated 10^5^ to 10^7^ species, more than any other single algal group [6,7]. A defining characteristic of diatoms is the frustule, which is composed of hydrated silicon dioxide and organic materials; environmental factors play a critical part in driving the morphological variation of frustules [8]. Diatom chloroplasts contain chlorophyll *a*, *c1*, and *c2*. The golden brown fucoxanthin, a promising antioxidant, anti-inflammatory drug, and anticancer drug, is a major carotenoid of diatoms [9]. Diatoms have been attracting increasing interest for their biological characteristics and application value. Due to their quantity, industrial scale culture conditions, as well as socio-economic considerations, diatoms are one of the most suitable raw materials for the production of bioactive metabolites. Progress on the identification of all of these components has recently been facilitated due to the development of new techniques, such as molecular genetic tools and different chemical approaches. However, aspects of diatom metabolism and the production of natural compounds still remain largely unknown.

## 2. Polyunsaturated Fatty Acids (PUFAs) and the Diatom

Certain indispensable fatty acids are important as components of the cell membrane, lipid storage or signal transduction pathways, for instance, polyunsaturated fatty acids (PUFAs). PUFAs are approximately 18–22 carbon, straight-chain fatty acids that contain two or more double bonds. PUFAs can mainly be classified into two principal families: *n*-6 (or ω-6) and *n*-3 (or ω-3) families that are derived biosynthetically from linoleic acids (18:2*n*-2) and α-linolenic acids (18:3*n*-3), respectively, and these two essential fatty acids (EFA) are crucial for human health. In the two families, *n*-3 PUFAs are represented by α-linolenic acid (ALA, 18:3*n*-3), stearidonic acid (SDA, 18:4*n*-3), eicosapentaenoic acid (EPA, 20:5*n*-3) and docosahexaenoic acid (DHA, 22:6*n*-3); *n*-6 PUFAs are represented by arachidonic acid (AA, 20:4*n*-6) and γ-linoleic acid (GLA, 18:3*n*-6). PUFAs as bioactive substances, especially the *n*-3 series, are essential nutrients that promote human health and growth in animals [10]. EPA and DHA play a favorable role in the cardiovascular system [11], vision [12], and treating psychiatric disorders [13]; while C18*n*-3 PUFAs do not confer such health benefits. Studies have shown that larger ratios of *n*-6 to *n*-3 PUFAs correlate with the increased pathogenesis of many diseases, including coronary heart disease (CHD) [14,15]. A convincing study by WHO/FAO demonstrated that consuming 250 mg (primary prevention) to 2 g (secondary prevention) of EPA and DHA every day can prevent CHD and reduce the risk of fatal CHD events (reviewed by Martins [16]). For human nutrition, DHA, an essential fatty acid, plays an important role in preventing age-associated declines in cognition, such as Alzheimer’s disease, multiple sclerosis and Parkinson's disease. DHA is also an active ingredient for the treatment of psychiatric conditions due to its function in nervous system development [17]. It must be emphasized that DHA can promote visual system development to enhance retinal function and visual acuity; particularly, preterm infants can benefit from an extra DHA supplement [18]. In aquaculture, EPA and DHA are the key nutritional constituents of the larvae of many fish, shrimp and bivalve organisms. PUFAs, which have the potential to maintain a high larval growth rate and high reproductive rate, are known in a wide manifold of marine and freshwater organisms [19,20,21]. Thus, PUFA content is one of the most important indices for the evaluation of aquatic feed nutritional value.

Due to the benefits of PUFAs in humans and animals, a large amount of PUFA supplementation is needed. However, the shortage of PUFA biological resources has always restricted their wide application; inexpensive DHA and EPA biological resources have become an urgent demand. It is generally recognized that the main sources of PUFAs on the market are marine fish because there are no reported definitive examples of land plants accumulating EPA and DHA [16]. Although the content of DHA and EPA in fish oil can reach approximately 20%–30%, there are many issues that remain to be solved. It must be emphasized that the quality of fish oil can vary much under different circumstances, for instance, fatty fish stemming from contaminated regions, those obtained during the fishing season, or the species of fish [22]. In addition, fish oil presents a disadvantage for some consumers due to its distinctive odor or off-favors that originate from lipid peroxidation [23].

Awareness of the benefits of PUFAs and the shortage of fish oil have led to the exploitation of new resources of PUFAs over the last few years. In actuality, fish are not real PUFA producers. Fish accumulate PUFAs through the ingestion of PUFA-rich microalgae. Microalgae, especially those of marine origin, as emerging resources of long-chain PUFAs, have caused increasing concern [24]. Lipid-rich microalgae have been regarded as promising candidates for the production of biofuels [25]. Diatoms are metabolically versatile species owing to the fact that they can produce and accumulate a range of bioactive metabolites, such as PUFAs and extracellular polymeric substances [1,26]. Using diatoms to produce PUFAs has more advantages than extraction from fatty fish. First, diatoms have a high content of PUFAs; the relative content of PUFAs in some species can reach up to 5%–6% of cell dry weight. Second, diatoms are single-celled microalgae with fast growth, and it is feasible to manipulate their metabolism to promote the synthesis of PUFAs using biotechnological means, such as genetic manipulation and modified culture conditions [27]. Thus, continuous mass production of diatoms using photobioreactors is easy, and highly stabilized metabolites can be obtained through controlled production conditions. Third, the extraction and purification of PUFAs from diatoms is much simpler than that from fatty fish [16]. In particular, diatom products do not contain a fishy smell, cholesterol, or pollution by pesticides and heavy metals that most likely appear in fish oil.

## 3. Environmental Factors Controlling the Synthesis of PUFAs

### 3.1. Light

More recently, microalgae biotechnologies have been explored intensively with the aim to increase fatty acid production. Light, one of the most important environmental factors of the marine ecosystem, is of increasing interest to modulate the growth and accumulation of fatty acids in microalgae. Microalgae biomass, fatty acid composition, and pigment and ester concentration are reported to be light sensitive, thus, much attention has been paid to enhancement of fatty acid production using variable light. Light, the source of energy for photosynthesis, can alter photosynthesis products to synthesize metabolites. The spectral property of light acts as a major factor in controlling photosynthetic efficiency. Blue and red light spectra have been revealed to be the most active for photosynthesis [28,29]. Studies of the effects of light on model diatom *Phaeodactylum tricornutum* suggested that *P. tricornutum* cultivated in tubular photobioreactors had decreasing biomass productivity under lowered light, whereas the EPA content showed increased production. Additionally, photo-inhibition, the phenomenon of excessively high irradiance on a culture, causes lower pigment content [30]. Studies on other microalgae also confirmed the correlation between cellular metabolism and light effects. For instance, Piepho *et al.* found that the interactive effects of light and phosphorus supply were most pronounced in the diatom *Cyclotella meneghiniana*, compared with the other two green algae *Scenedesmus quadricauda* and *Chlamydomonas globosa*, and a cryptophyte *Cryptomonas ovata*, and hypothesized that increasing light intensity can lead to higher production of total fatty acids (TFAs), saturated fatty acids (SFAs), and monounsaturated fatty acids (MUFAs), both at low phosphorus concentrations and high phosphorus concentrations in the centric diatom *Cyclotella meneghiniana* [31]. More distinct changes in several SFA and UFA (unsaturated fatty acid) concentrations with light were found in the low-P treatments compared with the high-P treatments. It is worth mentioning that in *Cryptomonas ovata* (Cryptophyceae), light intensity showed no effects on the production of TFAs, SFAs, MUFAs, and PUFAs [31]. In the range of maximum EPA productivity using *P. tricornutum* in a flat panel airlift reactor, photosynthesis efficiency reached 10.6% at a low light intensity (250 μmol photon m^−2^ s^−1^) [32]. Studies on stream periphyton provide some explanation regarding the changing of PUFAs production. These studies showed that the proportion of PUFAs decreased with increasing light intensity and increased with phosphorus enrichment, opposite of that observed for SFA and MUFA. In particular, under high phosphorus treatment, there was a significant augmentation of DHA. A decline in ALA under high light intensity was accompanied by an increase in linoleic acid under increasing light, while arachidonic acid was hardly affected by either light or phosphorus supply [33].

### 3.2. Temperature

Temperature plays a critical role in cell growth and metabolite synthesis. As the growth temperature changes, the responses from different species show inconsistent relationships between temperature and percentage of unsaturated fatty acid [34,35,36]. The optimum growth temperatures for some microalgae have been determined to range from 16 °C to 27 °C [28]; for example, the optimum temperature for maximum growth rate of *P. tricornutum* is 20 °C. The growth response of *P. tricornutum* was hardly affected by temperatures in short-term treatments, but in long-term temperature treatments, a gradual decrease of growth at lower temperatures and a sharp drop of growth rate at higher temperatures occurred [37]. The temperature experiment conducted on *Cyclotella* *meneghiniana* revealed that, TFA, SAFA, and MUFA concentrations increased at 25 °C compared to 10 °C under low P supply; while no difference was seen between 10 °C and 25 °C in the high-P treatment [31]. With regard to *Nitzschia closterium*, which has a curved relationship between temperature and PUFA content, its optimum temperature is 20–30 °C [34]. Marine benthic diatom species, separated from the intertidal environment, could accumulate PUFAs during low-temperature treatment, while short-chain fatty acids, glycerol and glucose were accumulated in response to high-temperature treatments [38]. The results are consistent with the observations in *Nitzschia frustulum* (Kützing) where a decreasing production of saturated and monounsaturated fatty acids and an increasing production of fatty acids with a high degree of unsaturation were detected at a low temperature treatment (10 °C) [34], which plays a critical role in the maintenance of membrane fluidity [39]. In *P. tricornutum*, which is considered to be a promising candidate for biodiesel production and is characterized by high fatty acid content, the contents of EPA and PUFAs were found to be increased by up to 120% compared with the control when temperature was lowered from 25 °C to 10 °C for 12 h, with PUFA and EPA yields of up to 4.9% and 2.6%, respectively [40].

### 3.3. Chemical Composition of Culture Medium

Diatoms are able to rapidly adapt to the changing nutrient conditions. Particularly, in upwelling water environments where nutrients are brought to the surface, diatoms exhibit notable efficiency in the uptake of growth-limiting nutrients such as iron, nitrogen and silica [41,42]. The chemical composition of culture medium (nitrogen, phosphorus, iron, silicon salt, vitamins) has considerable importance on the composition and content of fatty acids.

The type of nitrogen source can also affect fatty acid content and growth of microalgae. Diatoms are able to utilize a variety of nitrogen sources, including inorganic (NO_3_^−^, NH_4_^+^) and organic (urea, amino acids) nitrogen, thereby adapting their nitrogen metabolism based on the available nutrients [43,44]. Among three nitrogen sources, NaNO_3_, urea, and NH_4_Cl, tested in a lipid- and PUFA-rich pennate diatom *Cylindrotheca* species, the best growth of *C. fusiformis* occurred on nitrate and urea, while NH_4_Cl was best for *C. closterium* [45]. Lipid productivity was much higher in cultures supplied with NH_4_Cl for both *C. fusiformis* and *C. closterium* and compensated for the lower biomass in *C. fusiformis*. Thus, urea and NaNO_3_ are optimum nitrogen sources for growth of *C. fusiformis*, while NH_4_Cl is the best for *C. closterium*. The highest lipid content was achieved in both *Cylindrotheca* species in culture medium with NH_4_Cl as the nitrogen source. However, *C. fusiformis* showed a significantly lower growth rate in culture medium with NH_4_Cl than in medium with NaNO_3_ or urea [45]. Nitrate and urea are better than ammonium salts as nitrogen sources in *P. tricornutum* UTEX 640, a suitable strain for industrial production of EPA [32]. By using a flat panel airlift reactor, *P. tricornutum* biomass productivity reached 2.35 g L^−1^ day^−1^ on urea at an aeration rate of 0.66 vvm at continuous light supply (1000 μmol photon m^−2^ s^−1^), while productivity on nitrate never reached 1.37 g L^−1^ day^−1^ [32]. Many microalgae species present a higher lipid content under nitrogen starvation conditions, including diatom *P. tricornutum*, green algae *Chlorella* spp., *Botryococcus braunii*, *Chlamydomonas reinhardtii*, and *Dunaliella salina* [46,47,48,49,50]. Nitrogen deprivation leads to a redirection of intracellular carbon flux, and the carbon source is no longer converted to cellular building blocks but instead funneled into triacylglycerol synthesis in *P. tricornutum* [51].

Iron, a vital component of the photosynthetic apparatus and mitochondrial electron transport chain, is a growth-limiting nutrient for photosynthetic microalgae. Iron limitation was found to lead to reduced synthesis of chlorophyll and a significant decrease in photosynthetic efficiency as well as slower nitrogen assimilation in diatoms [52,53]. Vitamin B_12_ (cobalamin) availability is evidenced to influence diatom growth. The abundance and wide distribution of transcripts of CBA1, a recently characterized cobalamin acquisition protein, in environmental samples, suggests that vitamin B_12_ is an important nutritional factor [54].

In the weakly silicified diatom *P. tricornutum*, increasing the concentration of silicate was found to cause a decrease in the content of EPA [32]. In the marine diatom *Chaetoceros gracilis*, the content of EPA and DHA decreased as silicate availability was reduced [55].

### 3.4. Growth Stage and PUFA Accumulation

Usually, slow growth of microalgae leads to a high proportion of storage lipids, while higher growth rates result in higher fractions of structural lipids. PUFAs as parts of structural biomolecules are essential components of cell membranes. At low growth rates, microalgae tend to accumulate neutral lipids rich in SFA (14:0, 16:0) and MUFA (16:1). These lipids are used to store the excess carbon accumulated during photosynthesis. On the contrary, at high growth rates, the demand for structural components increases, thereby reducing the surplus for neutral lipid accumulation and consequently increasing the proportion of PUFAs [56].

*Cylindrotheca* strains, which are not usually used as food for marine organisms, accumulate a high percentage of PUFAs at the exponential phase (29.5%–42.9%), especially 20:5 (*n*-3) and 20:4 (*n*-6) [57]. A study on four strains of *Cylindrotheca* indicated that the total lipid content reached its highest values in the late stationary phase of the culture, while PUFAs decreased in the later phase and peaked in the exponential phase or in the early stationary phase [57]. This result is consistent with that of diatom *Thalassiosira pseudonana* by Brown *et al.* [58].

## 4. Growth Facilities

Growth facilities of diatoms can be set up in aquatic areas, which would not cause an arable land crisis. High-efficiency photobioreactors are required to culture and tap the potential of microalgae [59]. Photobioreactor systems mainly consist of two types: open and closed systems.

Open systems for the culture of microalgae, including open ponds and raceways, have been well developed. These open systems possess major advantages, including being of low cost and having simple technical requirements. Open systems can be used for fast-growing species or for species that can be maintained at extreme conditions, such as high salinity or high pH conditions, in which contamination can be controlled. A number of microalgae species have been commercially cultivated at large scale with open systems, such as green algae *Haematococcus pluvialis*, blue-green alga *Spirulina platensis*, *etc.* However, some issues need to be addressed, such as the likelihood of contamination from unwanted algae and certain micro-organisms, significant evaporation losses, poor utilization of CO_2_ due to evaporation or stripping, poor thermal or temperature control, and inability to achieve high-density cultures due to poor mixing and optically dark zones.

Closed photobioreactors have attracted much attention because they allow for better control of the culture conditions than open systems, thereby providing robust culture systems for the culture of a large number of microalgae species [60]. Higher biomass productivities can be achieved and contamination can be easily reduced by using closed photobioreactors [59]. The available photobioreactors of closed systems may be classified into tubular reactors and panel reactors. These can be further categorized according to the device used for light supply, the arrangement of the growth units, the orientation of tubes or panels, the method of gas exchange system, the method of culture circulation, and the materials used for photobioreactor construction [61]. Among these categories, irrespective of the specific reactor configuration, several criteria need to be addressed, including effective and efficient light supply; CO_2_ supply with minimal losses; continuous removal of oxygen generated by photosynthesis, as excessive oxygen inhibits photosynthesis and metabolism; and good scalability of the photobioreactor technology [62].

In both outdoor and indoor cultivation systems, light source and light intensity are key factors affecting the phototrophic growth of microalgae [63]. Despite the innovation of a large number of photobioreactors, only a few of them can effectively utilize solar energy for mass cultivation of microalgae [59]. Sunlight is the major light source for outdoor cultivation systems; innovative electric light sources, such as light emitting diodes (LED) and optical fiber, are of interest for indoor cultivation systems and 24 h/day production [64]. LED has high electrical-to-optical power conversion efficiency although its infrastructure investment is a bit more expensive. Most outdoor reactors are designed to have largely exposed illumination surface areas. In this respect, flat panel, inclined and horizontal tubular reactors are promising, except for their weak scalability. Reactors such as bubble-column, airlift, and stirred-tank are easy to scale up, although their use in outdoor cultivation is limited due to their small illumination surface area [59].

## 5. Genetic Engineering of Diatoms

Although large-scale cultivation facilities for microalgae are available, their productivity is hampered by the lack of ideal strains that can be selectively optimized for both high biomass productivity and high TAG (triacylglycerol) or PUFA content [25]. A promising approach is the use of genetic engineering to substantially improve the diatom strains to produce PUFA. Genetically modified crops, mammals, and microalgae are emerging as alternative sources of PUFA [65,66]. Genetically engineered microalgae represent progress toward the use of fermentation approaches to commercially exploit microalgae at large scale, thereby reducing the limitations associated with photobioreactors such as light-dependency and lower growth [67]. However, so far, genetic engineering of diatoms has been slow due to the lack of genetic transformation systems, and successful trials of genetically modified diatoms have been rarely reported.

**Figure 1:**
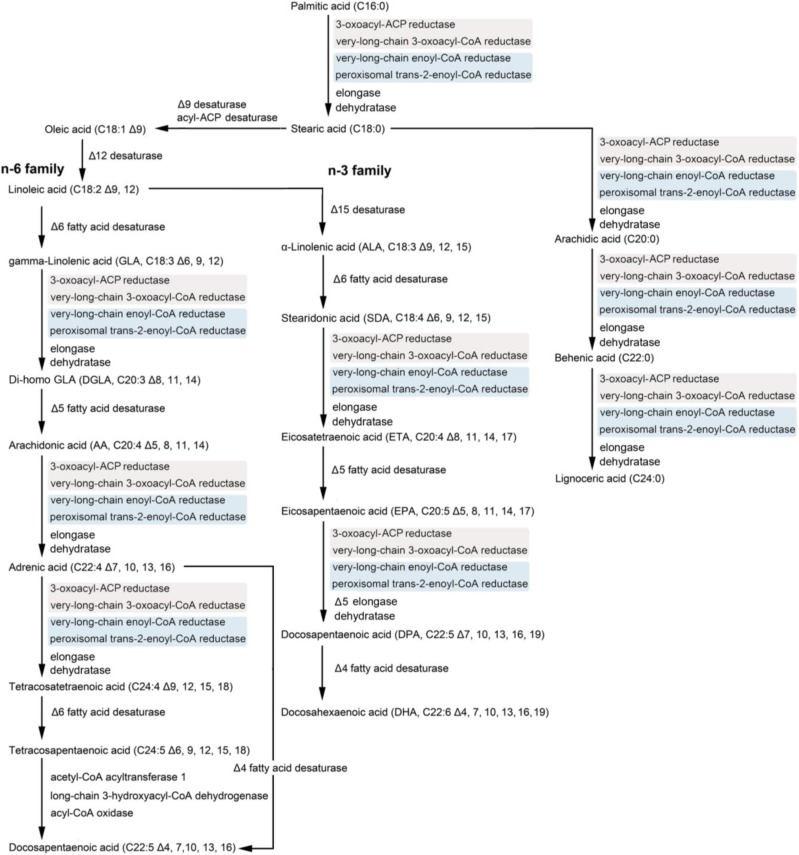
Proposed pathway of PUFA synthesis in diatoms.

With more and more sequenced genomes, certain metabolic pathways in the corresponding species can be readily proposed. By mapping annotated diatom genes to KEGG (Kyoto encyclopedia of genes and genomes) and taking the reported pathways into account [68,69], the PUFA biosynthetic pathway can be proposed in the diatoms *P. tricornutum* and *T. pseudonana* whose genome sequences are available (Figure 1). Those key points in the pathway are consequently the putative candidates for genetic engineering, as evidenced by studies in various organisms. By introducing a gene encoding a glucose transporter, transgenic *P. tricornutum* could thrive on exogenous glucose without light, thereby becoming heterotrophic [67]. This suggests that introduction of a single gene can lead to a fundamental change in the metabolism of a diatom.

Among the 11 open reading frames (ORFs) with significant similarity to fatty acid front-end desaturases in the genome sequence of *T. pseudonana*, two members are homologous to proteobacterial desaturases; one encodes a Δ11-desaturase active on palmitic acid; three encodes Δ4-, Δ5- and Δ6-desaturases involved in DHA production; and one encodes a Δ8-sphingolipid desaturase with substrate preference for dihydroxylated metabolites [70]. Heterologous expression of two *P. tricornutum* cDNAs encoding a microsomal and a plastidial Δ12-desaturase in yeast showed that the microsomal desaturase was suggested to be involved in the synthesis of EPA in the microsomes, while the plastidial desaturase showed high specificity for 16:1^Δ9^, indicating the plastidial origin of the hexadecatrienoic acid isomer (16:3^Δ6,9,12^) in *P. tricornutum* [71]. Δ5- and Δ6-fatty acid desaturases of *P. tricornutum* were characterized by expression in yeast, and the determination of substrate specificity of the two enzymes confirmed their role in EPA biosynthesis. Coexpression of both desaturases in combination with Δ6-elongase from a moss *Physcomitrella patens* reconstituted the biosynthetic pathways of arachidonic acid (AA) and EPA in yeast [72]. Bi-functional desaturases, such as Δ12/Δ15-desaturase from the amoebae *Acanthamoeba castellanii* and the fungus *Fusarium moniliforme*, have shown significant potential in increasing the productivity of *n*-3 substrates through redirection of the PUFA biosynthetic pathway [73,74]. It is worth noting that *n*-3 fatty acid desaturases are also functional enzymes in the synthesis of *n*-3 PUFAs, considering their roles for efficient conversion of *n*-6 PUFAs to *n*-3 PUFAs.

The pathway was proposed by mapping annotated genes to KEGG pathways based on the genomes of diatoms, including *P. tricornutum* and *T. pseudonana*. Isoenzymes are highlighted with the same color.

By heterologous coexpression of the Δ6-elongase from diatom *T. pseudonana* with Δ5-elongase, Δ5- and Δ4-desaturases from three other algae, DHA synthesis was successfully reconstituted in stearidonic acid-fed yeast [75]. PUFA biosynthesis could also be improved through the regulation of genes involved in lipid metabolism, for instance, overexpression of a Type 2 diacylglycerol acyltransferase which plays an important role in TAG assembly resulted in a 76% increase in PUFA content in *P. tricornutum* [76]. Moreover, fatty acid chain length could be regulated by genetic modification on certain genes in diatoms. Heterologous expression of plant acyl-ACP thioesterases biased towards the synthesis of lauric acid (C12:0) and myristic acid (C14:0) in *P. tricornutum* led to an increased accumulation of shorter chain length fatty acids [77]. Owing to the progress of functional genomics research together with the development and availability of molecular tools for diatoms [78,79,80], genetically engineering has been providing an effective way to improve the PUFA production phenotype of diatoms.

## 6. Conclusions

The large demand for health-benefiting PUFAs cannot be supplied by the current fish harvest. Therefore, new alternative sources of PUFAs have to be exploited. Using diatoms to produce PUFAs has several advantages, such as controlled culture conditions, lack of contamination, and the presence of PUFAs in the polar lipid fraction along with carotenoids, phytosterols, vitamins and antioxidants, which may also contribute to the health benefits of PUFA oils. Unfortunately, the production cost of autotrophic diatoms is still rather high. Nonetheless, further research is needed to create diatom strains with enhanced productivity of PUFAs. Enhancing the level of PUFAs is a major challenge, but reducing the *n*-6 and *n*-3 metabolic intermediates represents another important issue [65]. Genetically engineered diatoms that may be heterotrophically cultivated provide a new opportunity. Other limiting factors must also be taken into account, such as light, temperature, nutrients (iron, nitrogen, and silicon), pH, *etc.* for efficient industrial production of PUFA in diatoms.
